# Novel insights into plasma biomarker candidates in patients with chronic mountain sickness based on proteomics

**DOI:** 10.1042/BSR20202219

**Published:** 2021-01-19

**Authors:** Peili Zhang, Zhanquan Li, Faman Yang, Linhua Ji, Yingzhong Yang, Chuanchuan Liu, Huihui Liu, Jie Ma, Jie Liu, Zhancui Dang, Shengyan Wang, Rili Ge, Sen Cui

**Affiliations:** 1 Research Center for High Altitude Medicine, Qinghai University, Xining, China; 2 Key Laboratory of Application and Foundation for High Altitude Medicine Research in Qinghai Province, Xining, China; 3 Department of General Practice, Huadu District People’s Hospital, Southern Medical University, Guangzhou, China; 4 Department of Hematology, Affiliated Hospital of Qinghai University, Xining, China; 5 Department of Hematology, Huadu District People’s Hospital, Southern Medical University, Guangzhou, China

**Keywords:** biomarkers, chronic mountain sickness, hemoglobin beta chain, phosphoglycerate kinase 1, proteomics, thioredoxin-1

## Abstract

Chronic mountain sickness (CMS) is a progressive incapacitating syndrome induced by lifelong exposure to hypoxia. In the present study, proteomic analysis was used to identify the differentially expressed proteins (DEPs) and then evaluate the potential plasma biomarkers between CMS and non-CMS groups. A total of 145 DEPs were detected in CMS Han Chinese people who live in the plateau (CMS-HPu), among which 89 were significantly up-regulated and 56 were significantly down-regulated. GO enrichment analysis showed that various biological processes were enriched, including the hydrogen peroxide metabolic/catabolic process, reactive oxygen species (ROS) metabolic, and acute inflammatory response. Protein–protein interaction analysis showed that antioxidant activity, the hydrogen peroxide catabolic process and peroxidase activity were primarily mapped in interaction proteins. Nine modules showed significantly clustering based on WGCNA analysis, with two being the most significant, and GO analysis showed that proteins of both modules were primarily enriched in oxidative stress-related biological processes. Four DEPs increased in CMS patients were evaluated as the candidate biomarkers, and three showed significant AUC: hemoglobin β chain (HB-β), thioredoxin-1 (TRX1), and phosphoglycerate kinase 1 (PGK1). The present study provides insights into the pathogenesis of CMS and further evaluates the potentially biomarkers for its prevention and treatment of it.

## Introduction

Chronic mountain sickness (CMS) is a highly prevalent progressive incapacitating syndrome in most high-altitude regions around the world, that is defined by hemoglobin (Hb) concentration values ≥21 g/dl for men or ≥19 g/dl for women [1]. Many studies have shown that with the age and altitude, the prevalence of it increases significantly [2,3]. Meanwhile, as a clinical syndrome characterized by excessive erythrocytosis (EE) [4], CMS is usually accompanied by three or more of symptoms, including breathlessness, palpitations, sleep disturbance, cyanosis, dilation of veins, paresthesia, headache, or tinnitus, according to the Qinghai International Consensus scoring system [1]. It is estimated that 5–10% of the world’s population living at an altitude ≥2500 m above sea level may develop the condition [5–7]. Meanwhile, among people who live at an altitude >4000 m (e.g., the central Andes of Peru), up to 15–20% of the adult male population suffers from CMS, and its prevalence increases with age, reaching up to 27.4% by the age of 50 [2].

Generally, chronic hypoxic exposure is widely considered to be the potential inducement of CMS. The principal mechanism that the loss of ventilatory acclimatization to altitude hypoxia leads to central hypoventilation was proposed by Leon-Velarde [8] to explain accentuated hypoxemia and the subsequent EE response. In fact, hypoxic tissue conditions occur during a number of inflammatory diseases and are associated with the breakdown of barriers and induction of proinflammatory responses [9]. The blood supply and tissue metabolism were found to be disrupted in many pathological sites, which resulted in the tissue being exposed to unstructured and chaotic oxygen gradients [10]. Therefore, the exposure could lead to hypoxia, and then provoke pathophysiological responses, including inflammation and cell death, and finally contribute to the development of disease such as the CMS. On the other hand, low oxygen exposure at high altitudes increases the formation of reactive oxygen species (ROS) while decreasing the activity and effectiveness of antioxidant enzymes [11,12], which results in an increased level of ROS in the tissues. However, the specific mechanism that leads to CMS is still unclear. Therefore, in recent years, scientists around the world have made unremitting efforts to dissect the probable mechanisms using various technologies from a variety of unique perspectives to dissect the probable mechanisms, such as genomics [13–15] and proteomics [16].

In the present study, iTRAQ 4-plex technology was used to investigate the differentially expressed proteins (DEPs) between CMS patients and non-CMS participants, and bioinformatics was also used to excavate the potential target biomarkers in the development of CMS from a proteomic point of view. Our study demonstrated that various biological processes were changed in a low-oxygen environment, multiple candidate proteins associated with CMS were also evaluated, and the results indicated that they can be used as reliable indicators for CMS. Our study was also the first to demonstrate that HB-β, TRX1 and PGK1 can serve as possible biomarkers in patients with CMS.

## Materials and methods

### Subjects

The research protocol was approved by Ethics Committee at the Qinghai University Affiliated Hospital (Approval No. SL-2018098). Informed consent was obtained from each subject. Sixty participants were recruited for iTRAQ experiments, including 15 Han Chinese patients with CMS who lived at altitudes of 3400-4500 m (male; mean age 47.5 ± 11.4 years), 15 Han Chinese control participants who lived in a plateau at altitudes of 3400–4500 m, who were named HPu (male; mean age 51.1 ± 8.9 years); 15 native Tibetan control participants who lived in a plateau at altitudes of 3400–4500 m, who were named TPu (male; mean age 46.7 ± 12.9 years) and 15 Han Chinese control participants who lived in a plain at altitude of 20–30 m, who were named HPn (male; mean age 47.7 ± 10.4 years). The participants of CMS, HPu and TPu groups are from Yushu, Qinghai province of China, the geographical area of which is between longitude of E89°27′-E97°39′ and latitude of N31°45′-N36°10′, with an average altitude of 4493 meters. Whereas the participants of HPn group are from Nanjing, Jiangsu province of China, the geographical area of which is between longitude of E118°22′-E119°14′ and latitude of N31°14′-N32°37′, with an average altitude of 22 meters.

Fifty-seven participants were selected for enzyme-linked immunosorbent assay (ELISA) analysis, including 30 patients with CMS and 27 healthy controls (male; Han Chinese, with a similar lifestyle, living altitude and age distribution). None of the participants had a history of respiratory or cardiovascular disease, such as chronic obstructive pulmonary disease, asthma, infectious diseases, congenital heart disease or hypertensive heart disease. The presence and severity of CMS were judged by the ‘Consensus statement on chronic and subacute high-altitude diseases’ (Qinghai CMS score) [1].

### Sample preparation

The plasma was collected from the participants of four groups: (1) non-CMS Han Chinese people who lived in the plateau (nCMS-HPu); (2) CMS Han Chinese people who lived in the plateau (CMS-HPu); (3) the non-CMS native Tibetans people who lived in the plateau (nCMS-TPu), which served as the control in the present study, and (4) non-CMS Han Chinese people who lived in the plain (nCMS-HPn). Then, the plasma of all participants were centrifuged for 20 min at 3000 rpm, and the supernatant was stored at −80°C for further experiments.

The plasma used for ELISA analysis was prepared according to the following procedures. All the samples were incubated at room temperature for 20 min before centrifuging 20 min at 3000 rpm; then, the supernatant was transferred into a new tube, and the samples were stored at −80°C for further ELISA analysis.

### Protein extraction and peptides digestion

Protein extraction was carried out as described previously [17]. Briefly, 50 μl of plasma was added into the lysis buffer (4% [w/v] SDS, 100 mM Tris/HCl pH 7.6, 0.1 M DTT), vortexed for 30 s, and then centrifuged at 14,000 ***g*** at 4°C for 20 min. Protein quantification was performed based on the BCA method. The filter-aided sample preparation method was also used to perform peptides digestion [17]. Briefly, 100 μg of proteins were reduced with Tris-(2-carboxyethyl) phosphine (TCEP), alkylated with methyl methane-thiosulfonate (MMTS), and diluted 20 times before use. The proteins were then digested with trypsin at 37°C for 16 h with a final concentration of 10 ng/μl sequencing grade modified trypsin solution. Then, C18 cartridge was used to desalt the peptides after digestion. The peptide fractionations were dried using a vacuum freezer, and 40 μl dissolution buffer was then added into to redissolve. The peptide content was estimated by UV light at a spectral density of 280 nm using an extinction coefficient of 1.1 of 0.1% (g l^−1^) solution that was calculated on the basis of the frequency of tryptophan and tyrosine (the main UV light-absorbing amino acids at 280 nm) in vertebrate proteins [18].

### iTRAQ labeling

iTRAQ Reagent 4-plex Multiplex Kits were used to label each independent biological replicate (IBR) in the groups. For each group, 15 subjects were recruited, plasma samples of which were randomly mixed to three pools (five individuals for each IBR). Each iTRAQ reagent was diluted in 150 μl of isopropanol before 100 μg of peptide was added. Peptides were labeled as follows: Three IBRs of the nCMS-HPu group were labeled with iTRAQ tags 114; three IBRs of the CMS-HPu group were labeled with iTRAQ tags 115; three IBRs of the nCMS-TPu group were labeled with iTRAQ tags 116, and three IBRs of the nCMS-HPn group were labeled with iTRAQ tags 117. Next, 100 μl ultra-pure HPLC-grade water was added to terminate the labeling after the samples were labeled for 1 h. Samples were combined into one tube for each group, then resolved into 50 fractions using a 5-μm particle Ultremex SCX column (Phenomenex, Torrance, CA, U.S.A.), and finally desalted using a Gemini-NX C18 column (4.6 mm × 250 mm, Phenomenex). After all the fractions were freeze-dried under a vacuum, they were resuspended with 30 μl of mobile phase A (2% acetonitrile [ACN], 0.1% formic acid [FA]), divided into 15 groups based on their peak intensities and then centrifuged at 12,000 rpm for 10 min prior to LC-MS/MS analysis.

### LC-MS/MS analysis

A splitless nanoACQuity (Waters, Milford, MA, U.S.A.) system, including a pre-column packed with Symmetry C18 ((5 μm, 180 μm × 20 mm, Waters) and analytical column packed with BEH130 C18 (1.7 μm, 100 μm × 100 mm, Waters) was used in the present study. Then the AB SCIEX TripleTOF 5600 System (Concord, MA, U.S.A.) was used to analyze the samples, with the ion source being Nanospray III (AB SCIEX, U.S.A.) and the emitter being a needle drawn from quartz material (New Objectives, U.S.A.). Data were acquired using an ion spray voltage of 2.5 kV, curtain gas at 30 pounds per square inch (PSI), nebulizer gas at 15 PSI, and an interface heater temperature of 150°C. The MS was operated at a resolving power (RP) greater than or equal to 30,000 FWHM for the TOFMS scans (Agilent Technologies, Santa Clara, CA, U.S.A.). The survey scans were acquired at 250 ms, and as many as 30 product ion scans were collected when a threshold of 120 counts per second (counts/s) was exceeded, with a 2+ to 5+ charge-state for information-dependent data acquisition (IDA). The total cycle time was fixed at 3.3 s. The Q2 transmission window was set to 100 Da for 100%. Four-time bins were summed for each scan at a pulse frequency of 11 kHz by monitoring the 40-GHz multichannel TDC detector via four-anode/channel detection. A sweeping collision energy setting of 35 ± 5 eV coupled with iTRAQ-adjusted rolling collision energy was applied to all the precursor ions for collision-induced dissociation. The dynamic exclusion was set to 1/2 of the peak width (18 s), and then the precursor was refreshed off the exclusion list.

### Database search and protein quantitation

The Mascot search engine (Matrix Science, version 2.4.1) was used to identify the proteins against the human UniProt (20171211) sequence database. The criteria for the database search were set as follows: The digestion enzyme was set as trypsin with one missing cleavage at an MS/MS fragment ion mass tolerance of 0.1 Da and a peptide tolerance of 0.05 Da. The carbamidomethyl of cysteine, the iTRAQ 4-plex of lysine, and the N-terminal amino group of peptides were set as fixed modifications. Simultaneously, the oxidation of methionine and the iTRAQ 4-plex of tyrosine were set as variable modifications. The peptide charge was set as Mr and the monoisotopic mass was selected. The false discovery rate (FDR), which is the result of false positive matches divided by the total number of matches, accounted for <1.5% of the final results. Peptides that scored as significant (≥20) at a 99% confidence interval were used during the protein identification, and each confident protein included more than two confident peptides with at least one unique to this particular protein. For protein quantification, a median was introduced as the protein ratio type to demonstrate the reproducibility of the replicates, and then the ratios for each protein were normalized using log2. Based on previous studies [19,20], the fold change (FC) (FC > 1.2 or FC < 0.83) cutoff was used to identify DEPs with a global false discovery rate (FDR) of less than 5% in the present study. All mass spectrometry proteomics data were uploaded to proteomics database ProteomeXchange under the Acc. No. of PXD022139.

### Bioinformatics analysis

Gene ontology (GO) enrichment analysis was performed online using the Blast2GO software program (available online: http://www.geneontology.org) against UniProt. Pathway analysis was performed using the Kyoto Encyclopedia of Genes and Genomes (KEGG) (http://www.genome.jp/kegg/mapper.html). In addition, functional association networks of DEPs were constructed using STRING (http://string-db.org/), and ClueGo was used to perform a visualization analysis of the protein-protein interaction (PPI) [21]. Pheatmap (version 1.0.12), ggplot2 (version 3.0.0) and WGCNA (version 1.63) packages of R software (version 3.5.0) [22,23] were used for visualization of heatmaps, bubble diagrams and WGCNA analysis, respectively.

### ELISA analysis

ELISA kits for the hemoglobin β chain (HB-β, JL12856), protein S100-A8 (S100A8, JL33186), and thioredoxin-1 (TRX1, JL30796) were purchased from Shanghai Jianglai Biological Technology Co., Ltd.; the kit for phosphoglycerate kinase 1 (PGK1, JYM0585Hu) was purchased from Wuhan jiyinmei Biological Technology Co., Ltd. The formal experiments were performed following the manufactures’ instructions. The absorbance of each sample was detected at 450 nm using a TECAN Infinite M200 PRO (Schweiz).

### Statistical analysis

All data were analyzed using one-way ANOVA and were shown as means ± SEM; *P* values <0.05 were considered statistically significant, and SIMCA 14 was used to perform the principal component analysis (PCA).

## Results

### Clinicopathological phenotype of CMS

**Figure 1:**
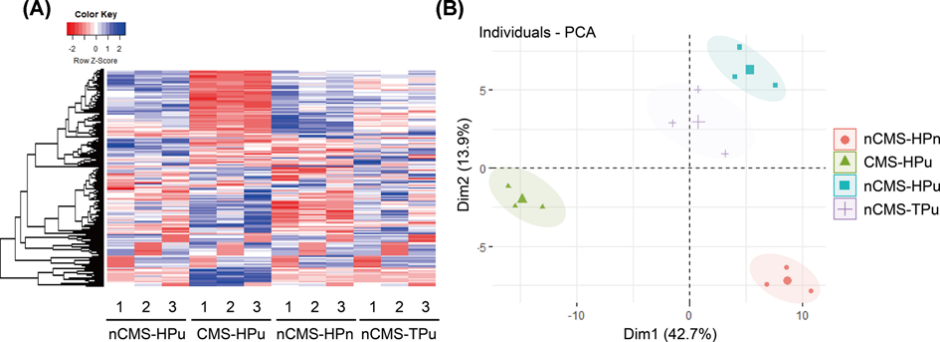
Heatmap analysis and principal component analysis (PCA) score plot for total proteins in four groups of plasma (**A**) The protein expression profile in each group with nCMS-HPu as reference. (**B**) PCA score plot in the plasma samples.

The general characteristics of the studied participants are shown in Table 1. There were no significant differences in age, height, body mass index (BMI), or blood pressure between the four groups. Compared with the three control groups, Hb (*P*<0.001), hematocrit (*P*<0.001), and erythrocyte counts (*P*<0.001) were significantly higher, while SaO_2_ was significantly lower (*P*<0.001) in the CMS group. The CMS score range was 7–16 points in patients with CMS. Therefore, iTRAQ 4-plex proteomic technology was then used to analyze the specific protein expression profiles among the four groups. As shown in Figure 1A, each group had a different protein expression profile of each group displayed different picture, especially the CMS-HPu group, whose profile was inverse to the other three groups, implying that the protein was closely related to the development of CMS. Here, a PCA score plot was also constructed to directly distinguish the expression profile. Four different parts were shown, suggesting that CMS altered the protein expression profiles (Figure 1B). Furthermore, the expression profile of nCMS-HPu was similar to that of nCMS-TPu which was consistent with the result of heatmap (Figure 1A,B).

**Table 1:** Clinical characteristics of the participants for the iTRAQ experiment

	CMS (*n*=15)	nCMS-HPu (*n*=15)	nCMS-TPu (*n*=15)	nCMS-HPn (*n*=15)	*P* value
Age, year	47.5 ± 11.4	51.1 ± 8.9	46.7 ± 12.9	47.7 ± 10.4	0.701
Height, cm	173.1 ± 4.6	172.4 ± 5.3	173.5 ± 4.6	171.7 ± 4.9	0.741
BMI, kg/m^2^	23.9 ± 2.3	3.7 ± 2.8	23.8 ± 2.6	24.7 ± 2.1	0.662
Systolic blood pressure, mmHg	125.5 ± 12.4	126.0 ± 17.4	126.4 ± 15.0	122.2 ± 15.7	0.869
Diastolic blood pressure, mmHg	81.0 ± 10.8	79.7 ± 8.5	79.5 ± 8.6	80.8 ± 11.7	0.965
Hemoglobin, g/l	228.5 ± 16.1	160.3 ± 11.3	164.6 ± 7.7	157.8 ± 6.1	<0.001
Hematocrit, %	68.1 ± 7.1	49.1 ± 3.2	49.0 ± 2.6	46.6 ± 1.8	<0.001
Erythrocyte, ×10^12^/l	7.4 ± 0.6	5.4 ± 0.4	5.4 ± 0.3	5.2 ± 0.3	<0.001
SaO_2_, %	88.5 ± 2.6	95.8 ± 1.1	95.9 ± 1.1	96.7 ± 1.5	<0.001
CMS score	11 (7-16)	2 (0-3)	2 (0-3)	1 (0-2)	<0.001

^1^Values are means ± SD unless otherwise specified; Abbreviations: CMS, chronic mountain sickness; SaO_2_, arterial O_2_ saturation.

### Identification of the differentially expressed proteins (DEPs)

**Figure 2:**
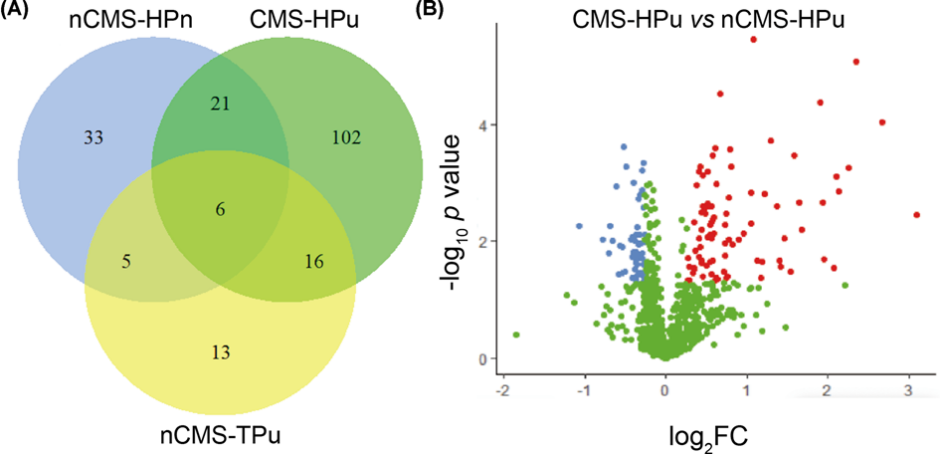
Venn and volcano plot analysis of the differentially expressed proteins (DEPs) compared with nCMS-HPu in three groups (**A**) Venn diagram; (**B**) volcano plot analysis of DEPs.

The potential pathogenesis of CMS was often associated with the key proteins. Therefore, DEPs were successfully identified based on the FC > 1.2 (or FC < 0.83) with *P*<0.05. There were 65 DEPs and 145 DEPs compared with nCMS-HPu in nCMS-HPn and CMS-HPu (Figure 2A), respectively. Among which, 27 DEPs were shared between both groups, and 38 DEPs and 118 DEPs were specific for nCMS-HPn and CMS-HPu (Figure 2A), respectively. Notably, 89 DEPs were significantly up-regulated and 56 DEPs were significantly down-regulated in CMS-HPu (Supplementary Table S1 and Figure 2B). In addition, 18 DEPs and 47 DEPs were significantly up-regulated and down-regulated, respectively (Supplementary Table S2 and Figure S1A), in nCMS-HPu compared with nCMS-HPn. Finally, 21 DEPs were up-regulated and 19 DEPS were down-regulated in nCMS-TPu compared with nCMS-HPu (Supplementary Table S3 and Figure S1B).

### GO enrichment analysis of CMS-HPu

**Figure 3:**
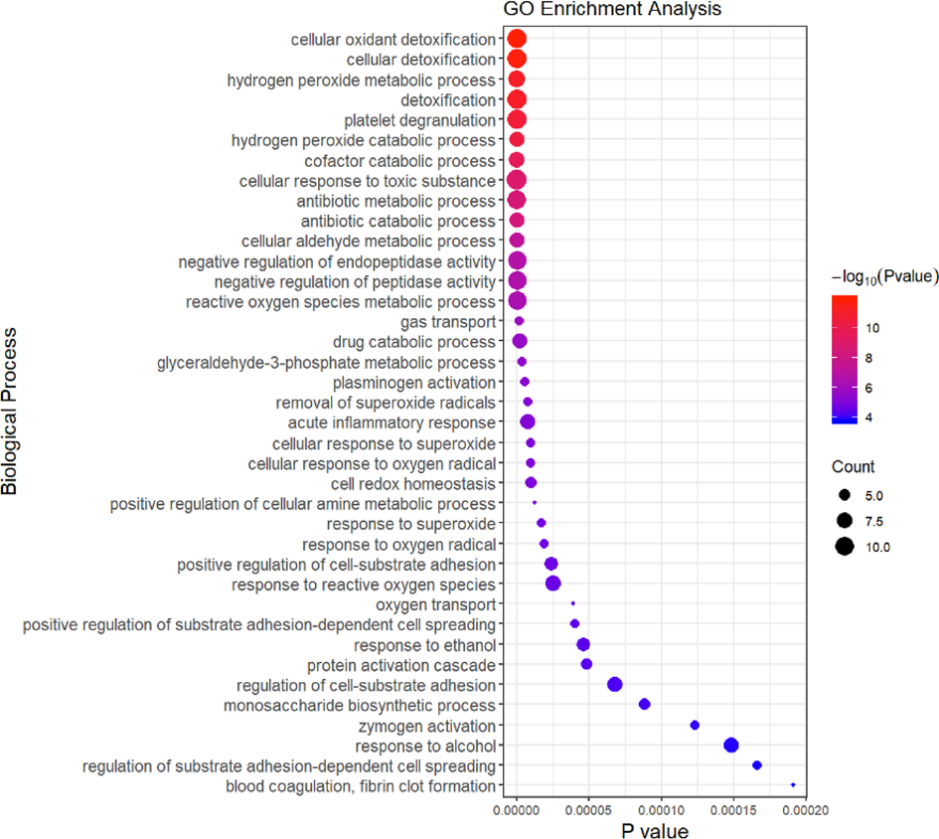
Biological process of GO enrichment analysis in the CMS-HPu group The size of the circle is determined by the number of proteins, and its color is determined by the *P* value.

GO enrichment analysis was performed and the results showed that the proteins involved in various biological processes were differentially accumulated in the CMS-HPu group (Figure 3; Supplementary Table S4). This finding showed that DEPs were significantly enriched for proteins linked to biological processes that were primarily focused on cellular oxidant detoxification, hydrogen peroxide metabolic/catabolic processes, platelet degranulation, cofactor catabolic process, antibiotic metabolic/catabolic processes, and reactive oxygen species (ROS) metabolic and acute inflammatory response (Figure 3), especially for ROS-related processes, suggesting that they played a critical role in regulating the development of CMS. Within the cellular components, the DEPs were primarily located in platelet alpha granule lumen and blood microparticles (Supplementary Figure S2 and Table S5). Finally, in terms of molecular function, the DEPs were primarily involved in the oxidoreductase activity, acting on peroxide as acceptors and antioxidants (Supplementary Figure S2 and Table S6). In conclusion, the redox process, including ROS metabolic, hydrogen peroxide metabolic/catabolic and inflammatory processes may be the key factors resulting in CMS. Functional annotation was also conducted for the DEPs between nCMS-HPn and nCMS-HPu groups (Supplementary Figures S3–S5). The DEPs were significantly enriched for proteins linked to biological processes that were primarily focused on platelet degranulation, negative regulation of response to wounding, negative regulation of blood coagulation and cell-matrix adhesion (Supplementary Figure S3). Within the cellular component, the DEPs were primarily located in blood microparticles and platelet alpha granules (Supplementary Figure S4). Additionally, in terms of molecular function, the DEPs were primarily involved in activity, such as peptidase regulator/inhibitor activity, antioxidant activity and endopeptidase regulator/inhibitor activity (Supplementary Figure S5).

### PPI analysis of the DEPs in the CMS-HPu group

**Figure 4:**
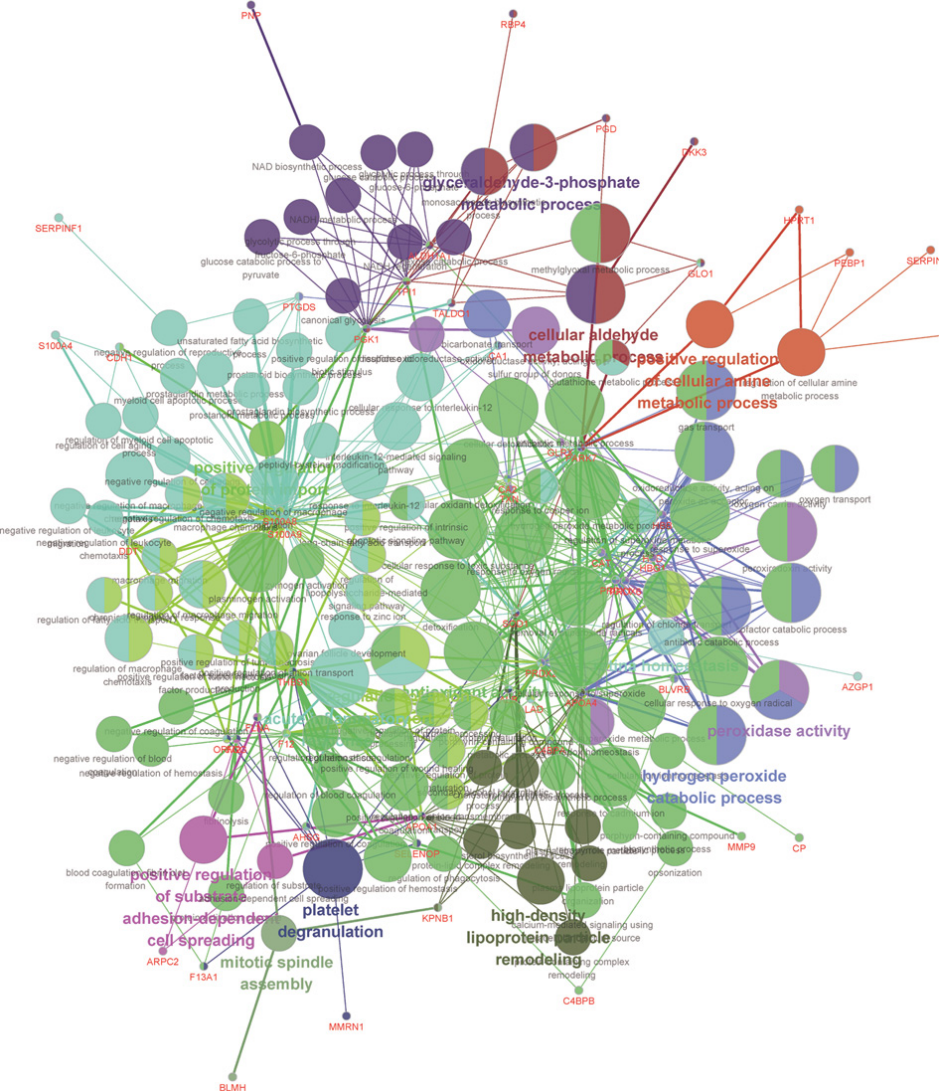
Protein–protein interaction (PPI) analysis of DEPs in the CMS-HPu group The node size represents the significance of DEPs, and the colors represent the different functional clusters.

To elucidate the interaction of the DEPs in the CMS-HPu group and confirm the function of interacting proteins, protein–protein interaction (PPI) analysis was conducted using ClueGo and the function for each module was also annotated. As shown in Figure 4, the proteins that interacted with the DEPs in the CMS-HPu group were primarily assigned to 14 functional modules. It is noteworthy that three of them were significantly associated with the redox process, including antioxidant activity, hydrogen peroxide catabolic process and peroxidase activity. Importantly, the acute inflammatory response process was also shown among the interacting proteins.

### WGCNA analysis of RNA-Seq in the CMS-HPu group

**Figure 5:**
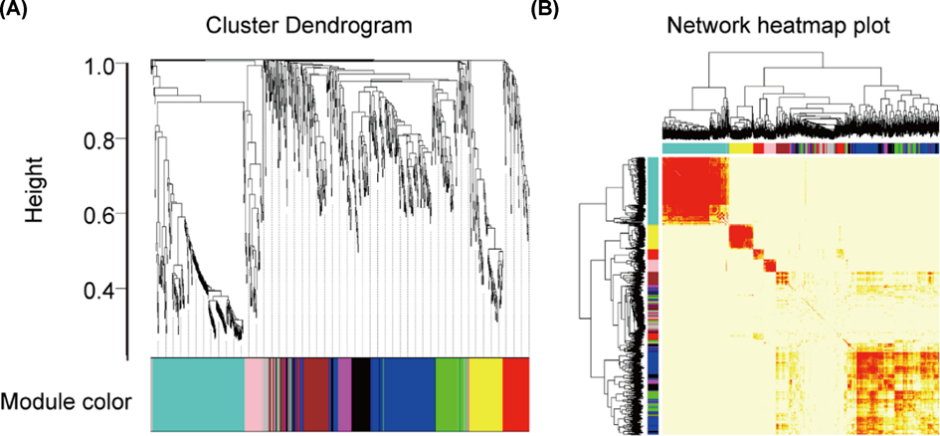
WGCNA cluster dendrogram and network heatmap plot of RNA-Seq in the CMS-HPu group (**A**) Dendrogram of modules identified by WGCNA. (**B**) topological overlap matrix among detected probes from RNA-Seq.

**Figure 6:**
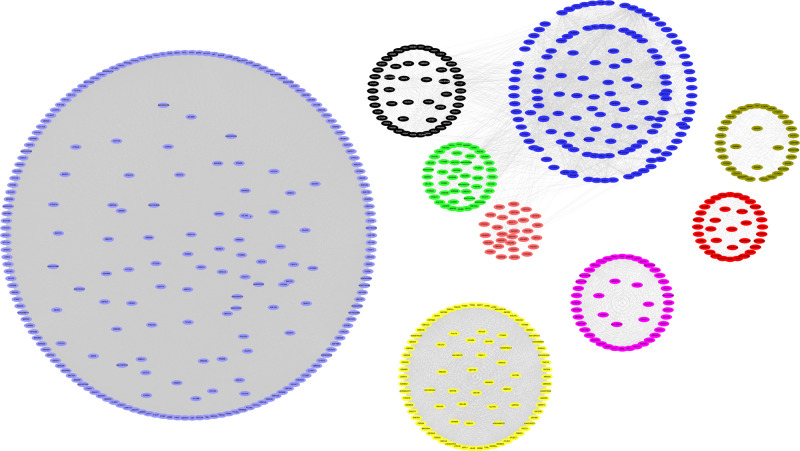
The significant modules of the WGCNA cluster in the CMS-HPu group

WGCNA is widely used to study co-expressed gene networks through scale-free weighted network construction and module detection [23]. To further confirm the gene network that was highly relevant to the protein expression in the CMS-HPu group, WGCNA was used to analyze the RNA-Seq. In the present study, 11 modules were successfully detected, and the gene clusters were displayed in a dendrogram (Figure 5A and Supplementary Table S7). In addition, the weighted network of identified genes from RNA-Seq was shown as a heatmap, which depicts the topological overlap matrix among all genes (Figure 5B). It is noteworthy that nine modules among them were showed significant clustering (Figure 6 and Supplementary Figure S6A), among which two modules, shown as black and magenta, were the most significant, with *P* values = 0.003 and 2e-05, respectively (Supplementary Figure S6A,B).

### GO analysis of the two most significant modules in the CMS-HPu group

**Figure 7:**
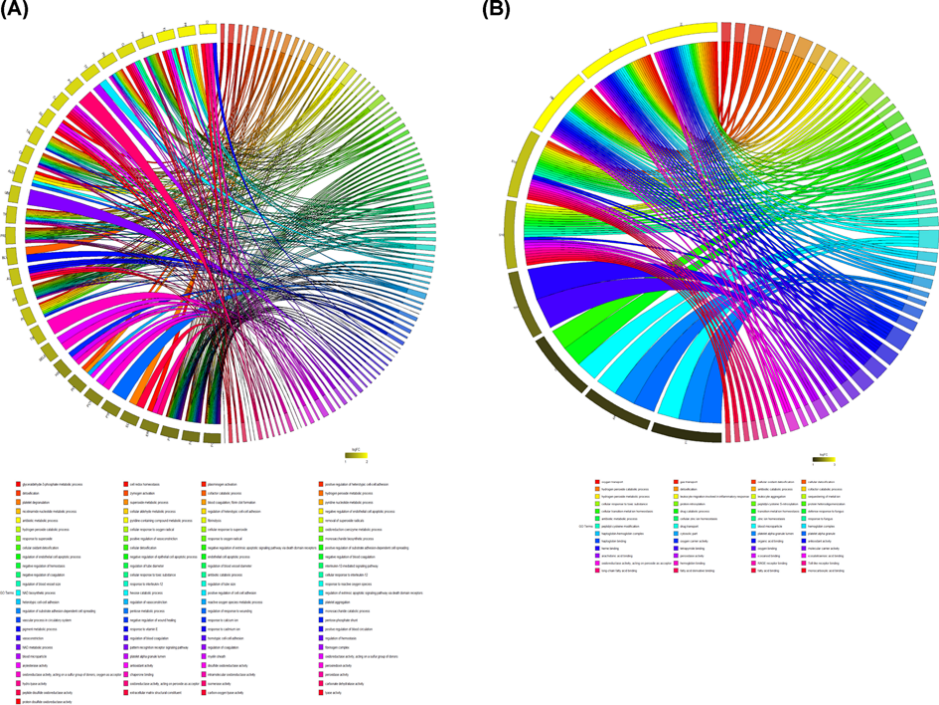
GO analysis of two most significant modules in the CMS-HPu group (**A**) The GO analysis of the black module. (**B**) The GO analysis of the magenta module.

To further identify the specific function of these two modules, GO annotation was then performed. The results showed that the black module’s DEPs were primarily differentially accumulated in the removal of superoxide radicals, hydrogen peroxide catabolic processes, cellular response to oxygen radicals, antibiotic metabolic processes, and oxidoreduction coenzyme metabolic processes (Figure 7A) among 97 associated processes. However, only 52 processes were annotated in the magenta-colored module (Figure 7B). Importantly, the DEPs, associated with the magenta module were primarily differentially accumulated in hydrogen peroxide metabolic processes, leukocyte migration involved in inflammatory response, leukocyte aggregation, and sequestering of metal ions.

**Figure 8:**
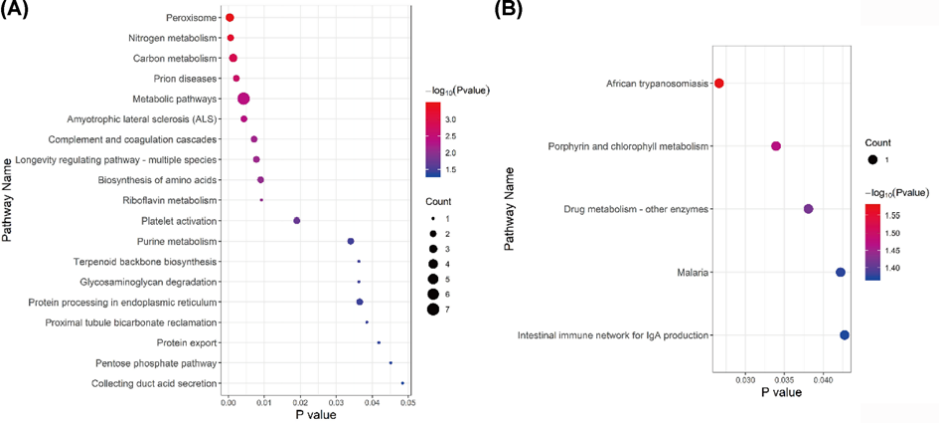
KEGG pathway analysis of two most significant modules in the CMS-HPu group (**A**) The pathway analysis of the black module. (**B**) The pathway analysis of the magenta module. The size of the circle is determined by the number of proteins, and its color is determined by the *P* value.

In addition, pathway analysis of these two modules was also performed (Figure 8). Within the black module (Figure 8A), proteins were primarily differentially accumulated in peroxisome, nitrogen metabolism, carbon metabolism, metabolic pathways, purine metabolism, and the pentose phosphate pathway (PPP). However, for the magenta module (Figure 8B), the proteins linked to KEGG pathways that were primarily focused on African trypanosomiasis and porphyrin and chlorophyll metabolism.

In short, redox and inflammatory processes were inseparable in the mechanism of CMS pathogenesis, and both may be potential strategies for the prevention and treatment of CMS.

### Evaluation of potential biomarkers in CMS and non-CMS participants

In the present study, CMS and non-CMS participants were recruited to evaluate the candidate biomarkers. The key physiological indicators were detected in the participants, and the results showed that three of them, namely hemoglobin, hematocrit and erythrocyte, were significantly increased in CMS compared with non-CMS participants (Table 2). However, the SaO_2_ was significantly reduced in CMS participants and the CMS-score was also higher than it in non-CMS participants.

**Table 2:** Characteristics of the participants for ELISA analysis

	CMS (*n*=30)	non-CMS (*n*=27)	*P* value
Age, year	46.8 ± 9.5	43.6 ± 9.6	0.207
Height, cm	172.8 ± 6.7	171.5 ± 6.3	0.437
BMI, kg/m2	24.3 ± 2.6	23.3 ± 1.9	0.093
Systolic blood pressure, mmHg	127.6 ± 19.6	122.1 ± 11.7	0.207
Diastolic blood pressure, mmHg	81.4 ± 16.3	77.3 ± 11.9	0.284
Hemoglobin, g/l	224.0 ± 14.4	155.3 ± 12.4	<0.001
Hematocrit, %	66.9 ± 4.8	46.3 ± 3.4	<0.001
Erythrocyte, ×10^12^/l	6.93 ± 0.5	5.1 ± 0.4	<0.001
SaO_2_, %	86.4 ± 3.7	94.3 ± 3.1	<0.001
CMS-score	11 (7-16)	2 (0-3)	<0.001

^1^Values are means ± SD unless otherwise specified. Abbreviations: CMS, chronic mountain sickness; SaO_2_, arterial O_2_ saturation.

**Figure 9:**
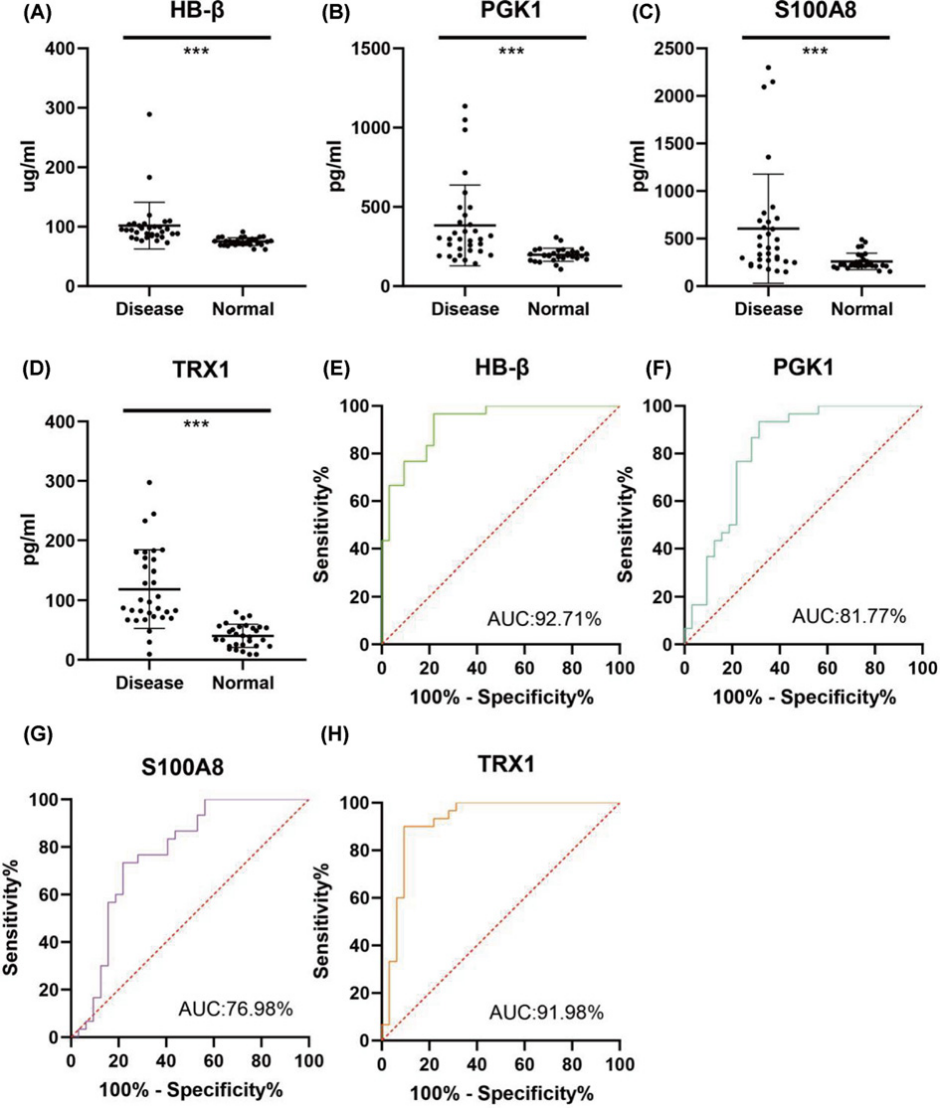
ELISA assay and ROC curve analysis between CMS and non-CMS participants Enzyme activity assays of HB-β (**A**), PGK1 (**B**), S100A8 (**C**), and TRX1 (**D**); ROC curve evaluation of of HB-β (**E**), PGK1 (**F**), S100A8 (**G**), and TRX1 (**H**). Data are presented as mean values ± *SD*; *n*=30-27; ***P<0.001.

Four DEPs were chosen from the most significantly enriched GO molecular function term for black module (TRX1 and PGK1 from GO:0016667, oxidoreductase activity, acting on a sulfur group of donors), and for magenta module (HB-β and S100A8 from GO:0043177, organic acid binding) for further ELISA validation. As a member of an ancient family of heme-associated proteins, iron-containing oxygen-transport metallo-protein in the red blood cells [24] is a major protein involved in the transport of oxygen, and the amount of HB-β is detected, together with PGK1, S100A8, and TRX1. As shown in Figure 9A–D, the activity of the four DEPs was significantly increased in the CMS group compared with the control, which was consistent with the levels of protein expression. Simultaneously, the area under the curve (AUC) of the receiver operating characteristic (ROC) curve demonstrated that all of the proteins mentioned above possesses high predictive and discriminatory capabilities (Figure 9E–H), especially HB-β, TRX1 and PGK1, suggesting that they could all be candidate evaluation indicators of CMS.

## Discussion

### Possible oxidative stress and inflammation in the CMS-HPu group

Our study focused on the plasma proteome characteristics of CMS-HPu, compared with non-CMS groups. A total of 145 DEPs were identified, which provided the most comprehensive proteomic information about CMS to date. WGCNA analysis identified two mous significant modules related to CMS. GO enrichment analysis of these modules showed that the hydrogen peroxide metabolic/catabolic process, reactive oxygen species (ROS) metabolism and acute inflammatory response, were enriched in the CMS-HPu group, indicating that inflammation and oxidative stress were closely related to CMS. Indeed, oxidative stress is caused by acute and chronic exposure to high altitudes. The low pressure and low oxygen concentration at plateaus are closely associated with oxidative stress reactions in organisms, and the compensatory process for quick adaptation to hypoxia greatly aggravates hypoxic oxidative stress reactions in cells and tissues [25,26]. Hypoxemia may be the upstream stimulus for oxidative catalysis, catalyzing not only systemic but also local free radical formation [27]. Notably, hypoxemia-induced systemic oxidative-nitrosative inflammatory stress was found to be markedly elevated in CMS compared with healthy HA (non-CMS) and low-altitude controls [28]. In conclusion, oxidative stress is increased following acute exposure to high altitude, and with chronic residence at high altitude.

Additionally, peroxisome, whose main function is to catalyze the β-oxidation of fatty acids and break down very long chain fatty acids (VLCFA) into short-chain fatty acids, was also enriched in our pathway analysis. A previous study showed that under chronic low oxygen conditions, PPP and one-carbon metabolism pathways were significantly enhanced in muscle to support the cytosolic redox balance and help mitigate the effects of increased protein and purine nucleotide catabolism in hypoxia [29]. Notably, PPP, carbon metabolism and purine metabolism were differentially accumulated in the CMS-HPu group, suggesting that peroxisome may play a critical role in developing hypoxia-related diseases, especially CMS, but the specific mechanism of action remains to be explored.

Meanwhile, some GO pathways that have not been reported involving in CMS were also enriched in our module analysis, such as glycosaminoglycan degradation and protein processing in endoplasmic reticulum (Figure 8A), suggesting new directions for future research.

### The potential biomarkers in the CMS-HPu group

In the present study, iTRAQ was used to identify the DEPs among the plasma samples of CMS-HPu, nCMS-HPu, nCMS-TPu and nCMS-HPn, and multiple DEPs were evaluated as the candidate biomarkers. Based on the WGCNA analysis, four proteins were chosen from two most significant modules and were evaluated via ELISA analysis for serving as potential plasma biomarkers. HB-β, a major protein involved in transport of oxygen, showed significantly increased levels of protein expression in CMS. Three major biochemical mechanisms have been put forward by researchers to explain the basis of free HB-mediated toxicity, including: (1) scavenging of endothelium-derived nitric oxide (NO), a vasodilator; (2) oversupply of oxygen; and (3) heme-mediated oxidative reactions [30]. The interactions of haptoglobin (Hp) with HB in particular may explain the molecular basis of Hp’s action in protecting HB against its own oxidative toxicity in circulation. In fact, the hotspots amino acids (i.e., βCys93) of HB were amenable to oxidation; however, Hp has been shown to allow the heme active site to operate unhindered leading to the elimination of oxidants [31]. Meanwhile, a tyrosine residue at the HB α subunit (αTyr42) acts as a conduit for electron transfer to and from the heme which facilitates the auto-reduction of the ferrylHB [32]. Studies have also shown that this electron transfer is diverted to another Tyr residue, namely βTyr145, when HB is complexed with Hp in the presence of H_2_O_2_ in a process that stabilizes the ferryl-induced free radicals on βTyr145, indicating that radical reactivity may ultimately be directed to the Hp molecule resulting in a safer redox inactive HB molecule [33]. Additionally, it was confirmed HB-β is enriched in pyramidal neurons in internal layers of the cortex and interacts with multiple proteins, which are located in neurons and mitochondria, to provide neuroprotection by supporting neuronal metabolism [34].

TRX1 is also involved in the regulation of redox homeostasis; its major role is to donate a hydrogen atom to enzymes involved in reductive reactions [35,36]. Given the anti-inflammation and antioxidation properties of Trx-1, a previous study showed that overexpression of Trx-1 can significantly suppress the NF-κB inflammatory signal pathway by inhibiting the activation of molecules involved in endoplasmic reticulum stress [37]. It was demonstrated that adult mice overexpressing Trx-1 had increased resistance to oxidative stress and reduced oxidative damage to proteins and lipids [38], suggesting the key role of Trx-1 in regulating ROS.

As an ATP-generating enzyme of the glycolytic pathway catalyzing the conversion of 1,3-diphosphoglycerate to 3-phosphoglycerate, and as a hypoxia-inducible factor, the expression of PGK1, whose induction is fully abolished when silencing of hypoxia-induced factor-1α (HIF-1α)—which is the most important factor involved in the cellular response to hypoxia [39]—was significantly upregulated under hypoxia [40]. This strengthens the perspectives for additional novel therapeutic approaches targeting hypoxia-dependent factors.

It is believed that S100A8 is closely involved in inflammation [41,42], and it has previously been identified as a proinflammatory factor in arthritis and autoimmune disease [43]. Measurement of the plasma levels of S100A8, one of the principal mediators of the innate immune response, has been proposed as diagnostic marker for several inflammatory conditions [41]. Importantly as an inflammatory protein, it is observed that its levels were found to be significantly elevated [43]. Additionally, a previous study demonstrated a correlation between the expression levels of protein S100A8 and inflammation via the Wnt/β catenin signaling pathway, implying a role in proinflammatory role [44]. In general, the four potential biomarkers might provide a direction for novel anti-inflammatory or anti-oxidative stress, and all of them could be effective biomarkers for CMS, especially HB-β, TRX1, and PGK1.

In summary, proteomic analysis was used to identify the DEPs and then validate the potential plasma biomarkers for CMS. A total of 145 DEPs were detected, among which 89 were significantly up-regulated and 56 down-regulated. GO enrichment analysis showed that the hydrogen peroxide metabolic/catabolic process, reactive oxygen species (ROS) metabolic and acute inflammatory response were significantly enriched. Protein–protein interaction analysis showed that antioxidant activity, the hydrogen peroxide catabolic process and peroxidase activity processed the key nodes in the networks. WGCNA analysis identified nine modules, with two being the most significant. GO enrichment analysis showed that proteins of both modules were primarily enriched in oxidative stress-related biological processes. Four DEPs were chosen from the most enriched GO terms for both modules, and were evaluated as the candidate biomarkers by ELISA. Three proteins showed significant AUC: hemoglobin β chain (HB-β), thioredoxin-1 (TRX1), and phosphoglycerate kinase 1 (PGK1). Our work provided insights into the proteome profiles of CMS and further evaluated potentially biomarkers for prevention and treatment of CMS.

## Supplementary Material

Supplementary Figures S1-S6

Supplementary Tables S1-S7

## Data Availability

All proteomic data were deposited and available in database ProteomeXchange under the Acc. No. PXD022139.

## Abbreviations

CMS: chronic mountain sickness
DEP: differentially expressed protein
EE: excessive erythrocytosis
HB-β: hemoglobin β chain
PGK1: phosphoglycerate kinase 1
ROS: reactive oxygen species
TRX1: thioredoxin-1
