# Supplementary material for: Novel insights into plasma biomarker candidates in patients with chronic mountain sickness based on proteomics
Source: Biosci Rep. 2021 Jan 19;41(1):BSR20202219. doi: 10.1042/BSR20202219 (PMC7816071; doi:10.1042/BSR20202219)
Supplement: Supplementary Figures S1-S6 [file BSR-2020-2219_supp.pdf]

Figure S1

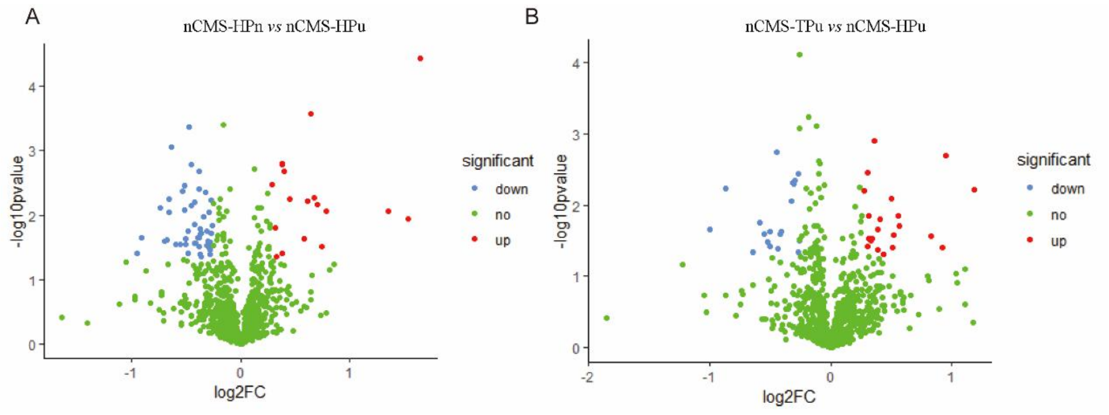

Figure S1 nCMS-HPn/nCMS-HPu and nCMS-TPu/nCMS-HPu volcano Plot

Figure S2

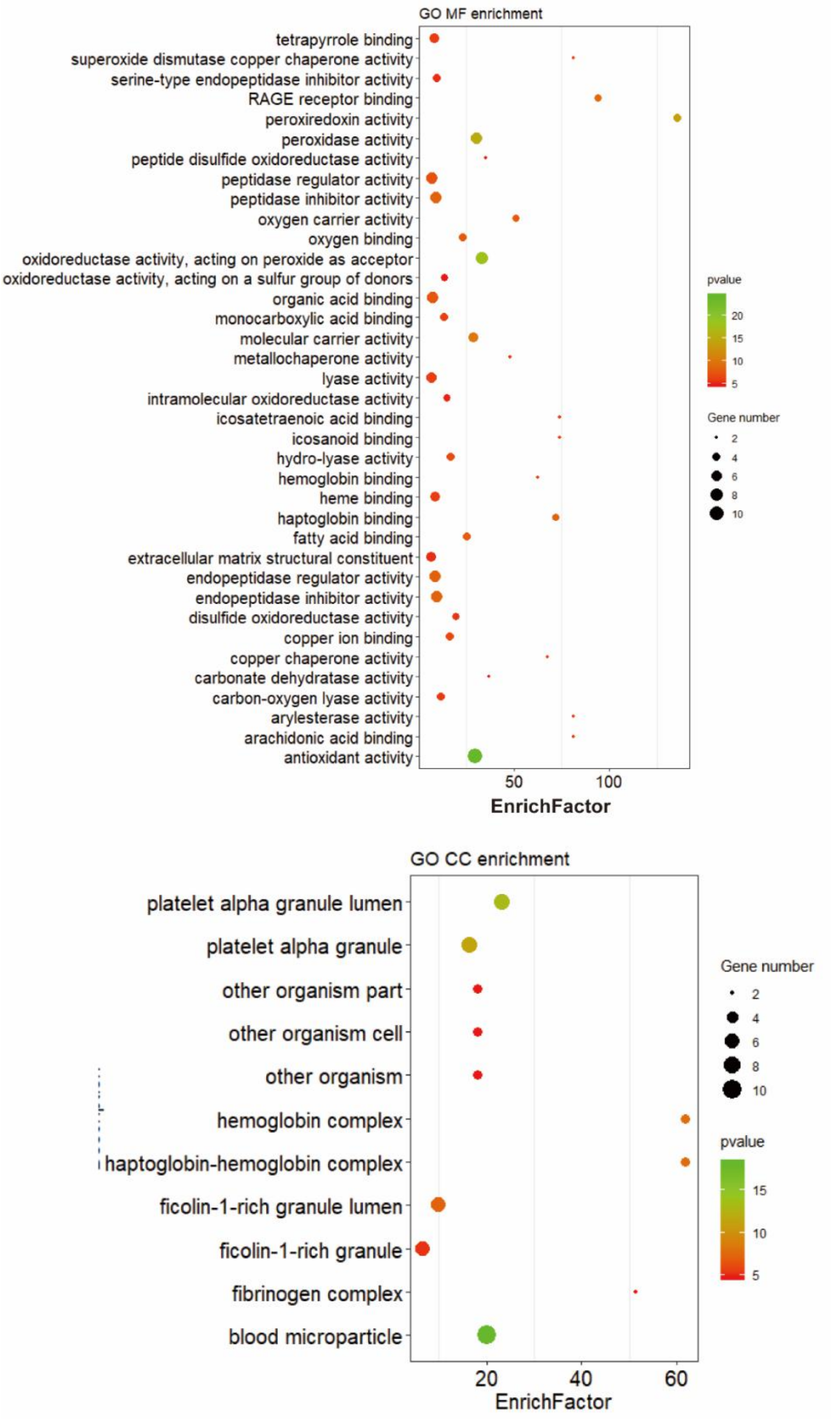

Figure S2 GO biological process and molecular function of CMS-HPu/nCMS-HPu

Figure S3

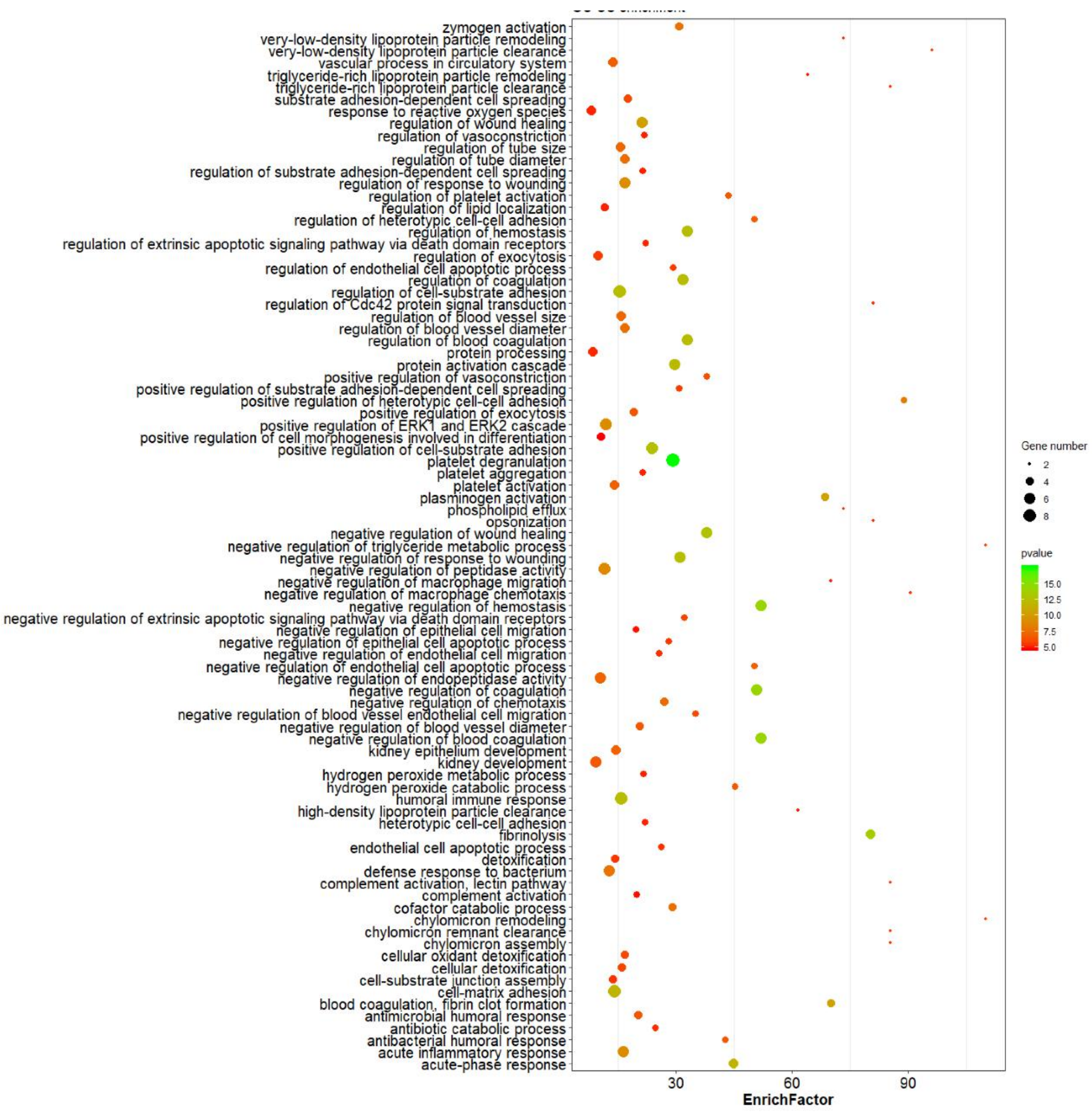

Figure S3 GO biological process of nCMS-HPn/nCMS-HPu

Figure
S4

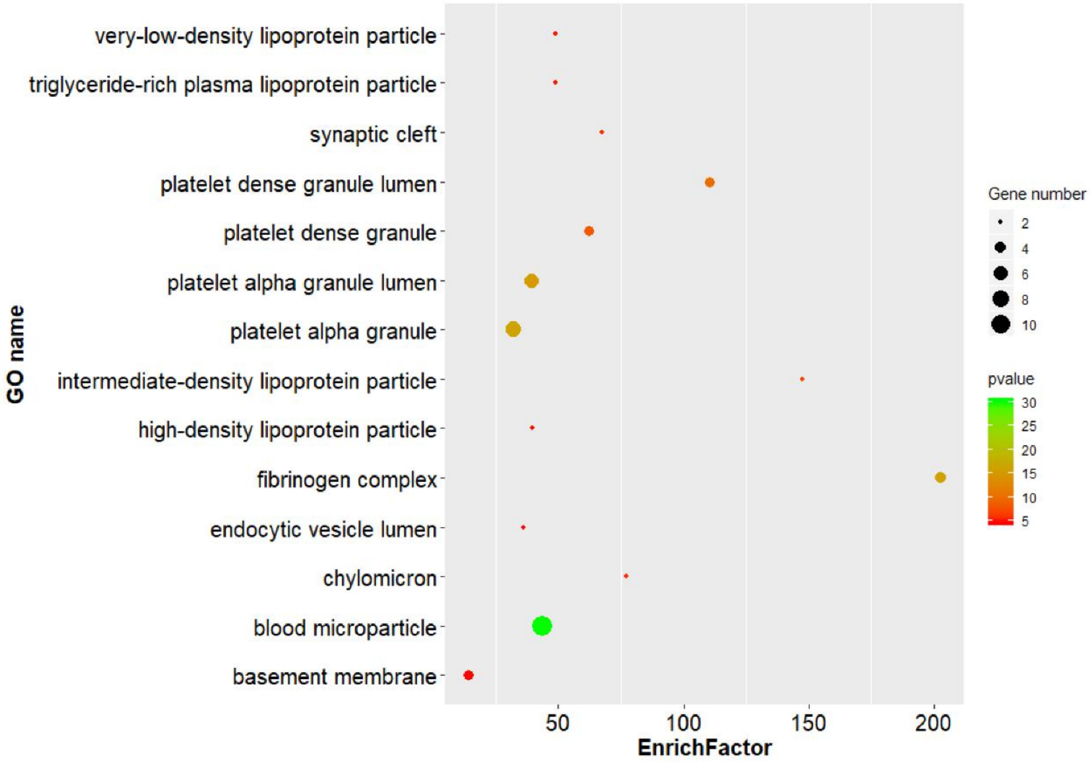

Figure S4 GO cellular component of nCMS-HPn/nCMS-HPu

Figure S5

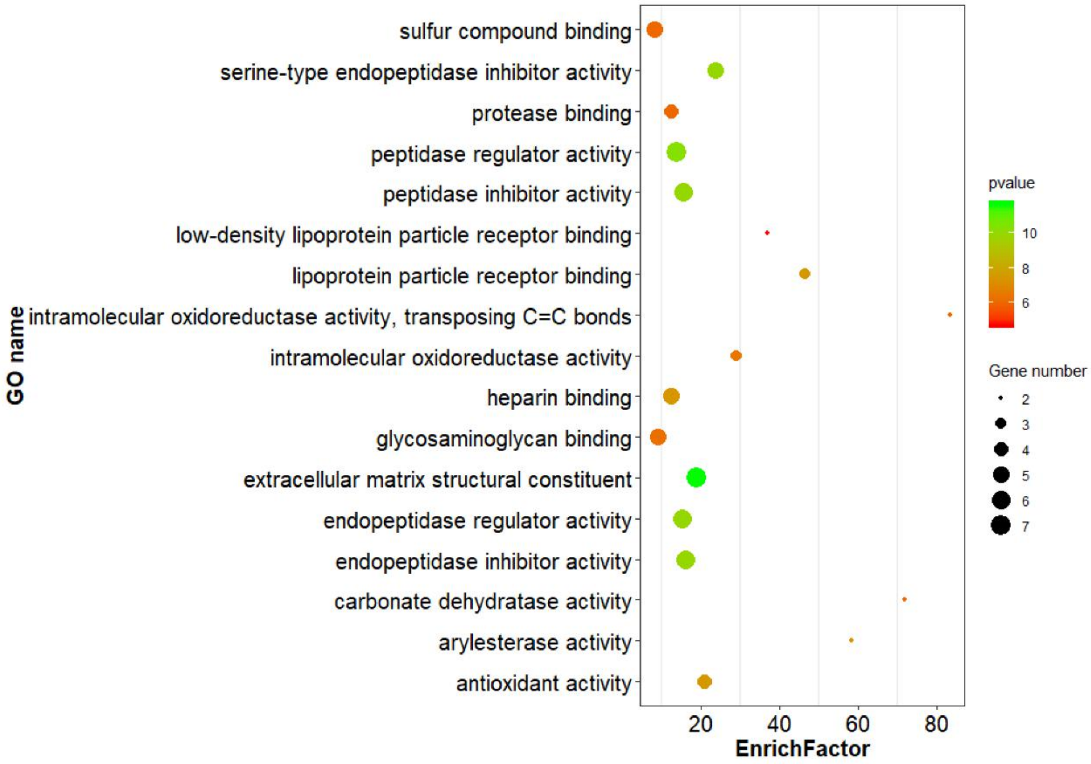

Figure S5 GO molecular function nCMS-HPn/nCMS-HPu

Figure S6

A

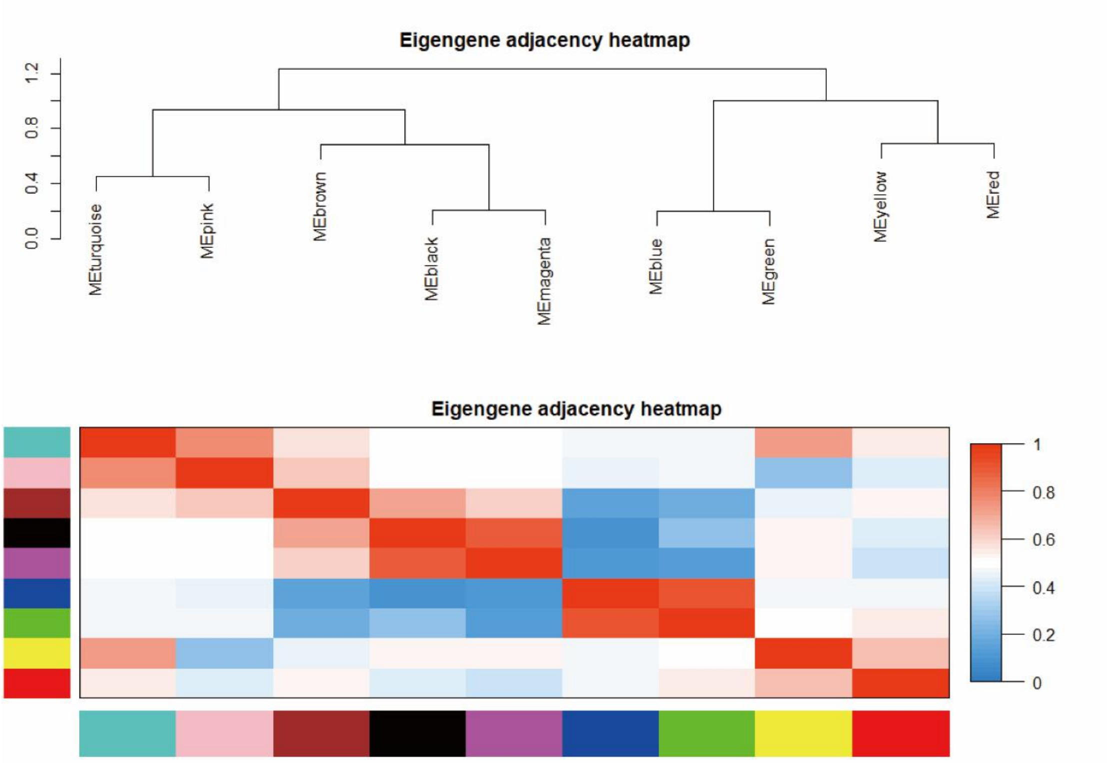

B

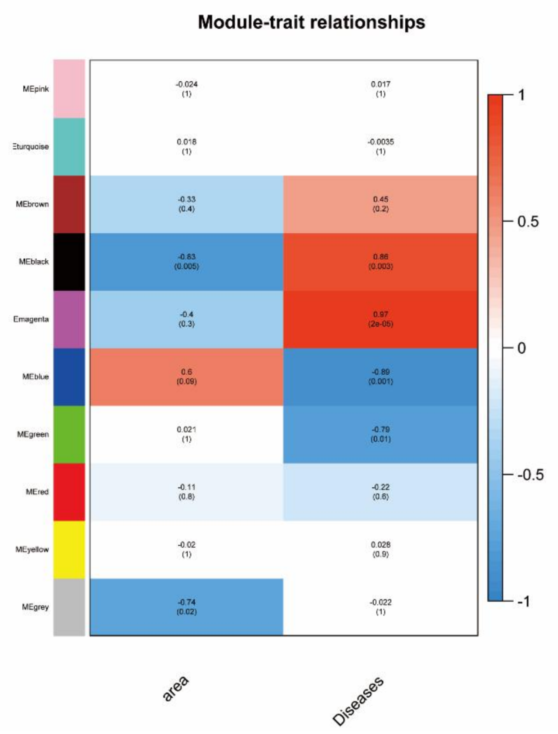

Figure S6 Eigengene adjacency heatmap and Module-trait relationships
